# Clinical use of HD-MTX monotherapy in a rare case of refractory primary bone diffuse large B-cell lymphoma with long-term survival after local radiotherapy: A case report

**DOI:** 10.3389/fmed.2022.989590

**Published:** 2022-12-21

**Authors:** Zhiya Wu, Yue Teng, Jianqiu Wu, Honglu Zhang, Weiwei Peng, Cheng Meng, Weiyan Tang, Jifeng Feng

**Affiliations:** ^1^Department of Oncology, Jiangsu Cancer Hospital and Jiangsu Institute of Cancer Research, The Affiliated Cancer Hospital of Nanjing Medical University, Nanjing, China; ^2^School of Basic Medicine and Clinical Pharmacy, China Pharmaceutical University, Nanjing, China

**Keywords:** methotrexate, primary bone lymphoma, primary bone diffuse large B-cell lymphoma, refractory, case report

## Abstract

Primary bone diffuse large B-cell lymphoma (PB-DLBCL) has been rarely reported because of its low incidence. The optimal treatment plan for patients with relapsed/refractory PB-DLBCL remains controversial. In this study, we present a case of a 57-year-old patient with refractory PB-DLBCL to better understand this disease. The patient developed lumbosacral/low extremity pain. A lumbar magnetic resonance imaging (MRI) revealed skeletal lesions with osteolysis in L4-L5 and S1. With the failure of multi-line chemotherapy, the patient developed paralysis of both lower limbs. 18-Fluorinefluorodeoxyglucose positron emission tomography/computed tomography (18F-FDG PET/CT) and MRI showed new lesions in the femoral head, cervical and thoracic vertebrae. We tried to treat the patient with adjuvant radiotherapy and 10 courses of high-dose methotrexate (HD-MTX)-based monotherapy, after which the patient was able to walk and achieved complete remission (CR). To the best of our knowledge, this is the first attempt to use local radiotherapy combined with an HD-MTX regimen successfully for the treatment of refractory PB-DLBCL.

## 1 Introduction

Bone lymphoma can be divided into three types: primary bone lymphoma, consisting of a single bone lesion with or without regional lymph node involvement; polyostotic lymphoma, consisting of multiple bone lesions without lymph nodal or visceral disease; and disseminated lymphomas with secondary involvement of the skeleton (1). The most common pathology of primary bone lymphoma (PBL) is DLBCL, and the less common pathologies are follicular, anaplastic large-cell, Hodgkin, marginal zone, and T-cell lymphomas (2). The clinical manifestations of PBL include bone pain, palpable mass, fractures, and neurological symptoms. The spine is the most frequently affected site. Prior studies have shown that patients with PB-DLBCL have a favorable prognosis in comparison with those with other extranodal lymphomas. PBL patients whose primary tumor sites in the axial skeleton have worse survival than those with primary tumor sites in appendicular and craniofacial skeletons (3).

The median age of patients with PB-DLBCL is around 55 years (4, 5). PBL is rare, accounting for only 5% of all extranodal primary lymphomas and <1% of all non-Hodgkin lymphomas (NHL). PBL lacks specific clinical and imaging features. Moreover, it seems impossible to conduct large prospective clinical trials in PBL due to its low incidence. Therefore, the best treatment option for PBL has not been found.

In order to enrich the experience in the treatment of PBL, we report a case of PB-DLBCL on HD-MTX monotherapy after multi-line treatment failure, through which the patient recovered from paralysis and achieved long-term survival for more than 10 years.

## 2 Case report

A 57-year-old Chinese woman (155 cm, 60 kg, 1.58 m^2^) presented with lumbosacral/low extremity pain for 2 months. The patient did not have any history of lumbar disk herniation or fracture. Her family history was also negative. Before presenting to our hospital, she had received treatment in several institutions. Initially, she had visited a local hospital and had received acupuncture and moxibustion, which showed no efficiency. A lumbar MRI revealed an abnormal signal in L4, L5, and S1, accompanied by soft tissue shadows in the paravertebral and the spinal canal. Previously, in other hospitals, the patient had received empirical anti-tuberculosis treatment for 4 weeks, which showed poor efficiency. She developed a new symptom of cauda equina syndrome (CES) later. To her relieve the pain and determine the pathology, we performed disk decompression and a biopsy of paralumbar soft tissue. We carried out immunohistochemistry tests to determine the pathology, which indicated that tumor cells were positive for LCA, CD20, CD79, CD10, and BCL-2; were negative for CD3, CD56, MUM-1, CD138, and CD99; and showed a Ki-67 proliferation index of greater than 80%, which is a characteristic of DLBCL. The diagnosis was reconfirmed by the pathologists in our institution. Bone marrow aspiration came out negative and the echocardiogram appeared normal.

Initially, the patient was administered one cycle of the CHOP regimen (cyclophosphamide 750 mg/m^2^, doxorubicin 50 mg/m^2^, vincristine 2 mg, day 1, and prednisone 100 mg, days 1–5, administered every 21 days) and six cycles of the R-CHOP regimen (rituximab 375 mg/m^2^, cyclophosphamide 750 mg/m^2^, doxorubicin 50 mg/m^2^, vincristine 2 mg, day 1, and prednisone 100 mg, days 1–5, administered every 21 days). The average relative dose intensity (RDI) was 100%. One month after the last chemotherapy, lumbosacral pain recurred. 18F-FDG PET/CT showed diffuse hypermetabolic foci in C2, C3, and C5 (SUVmax 13.5) and T11-L2 (SUVmax17.8). Considering the response evaluation of the R-CHOP regimen showed progressive disease (PD), local doctors changed one course of the MA regimen (methotrexate 1 g/m^2^, day 1 and cytarabine 3 g/m^2^, 12 h apart, days 2–3, administered every 21 days) and two courses of DHAP regimens (dexamethasone 40 mg, days 1–4, cisplatin 100 mg/m^2^, day 1, and high-dose cytarabine 2 g/m^2^, 12 h apart, day 2, administered every 21 days), both of which were unsuccessful.

**FIGURE 1:**
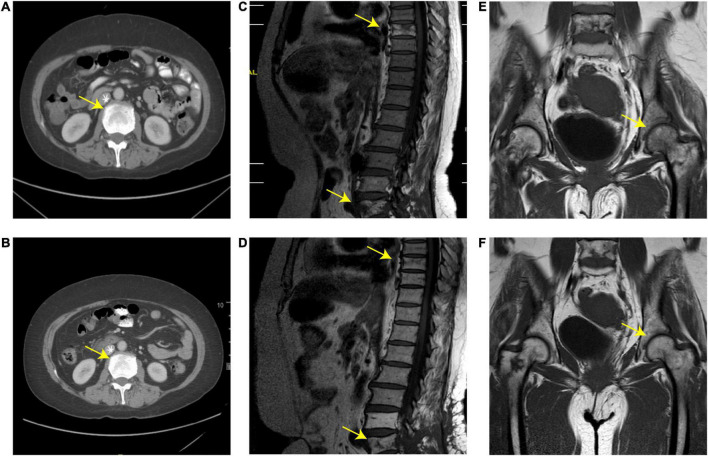
**(A,B)** Abdominal computed tomography (CT) scan; **(C,D)** spine magnetic resonance imaging (MRI); **(E,F)** MRI of the hip joint before and after treatment. **(A,C,E)** Skeletal lesions with osteolysis in the femoral head, T8, and L4-L5 before local radiotherapy. **(B,D,F)** Osteolytic lesions diminished after local radiotherapy and high-dose methotrexate (HD-MTX) treatment. The yellow arrows represent the location of the lesion.

**FIGURE 2:**
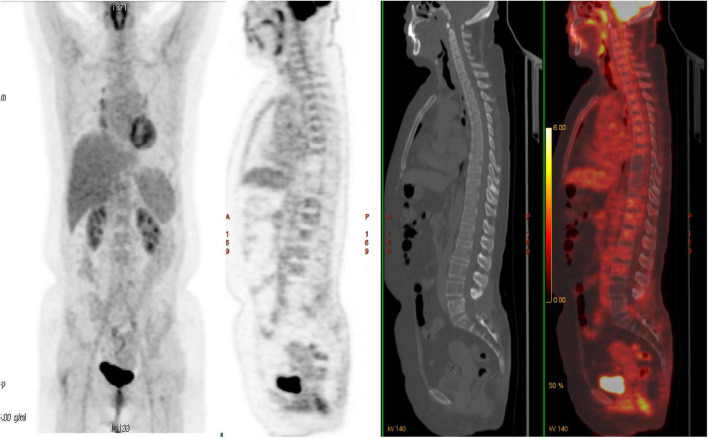
Follow-up fluorodeoxyglucose positron emission tomography/computed tomography (FDG-PET/CT) after high-dose methotrexate (HD-MTX) showed complete metabolic response with the spine. The patient has maintained complete remission for 10 years after the initial treatment.

**FIGURE 3:**
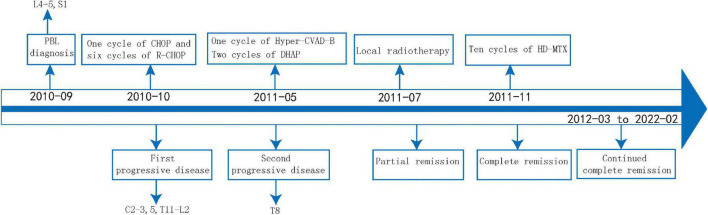
Treatment timeline of the patient with PBL.

Then, the patient presented to our department. She suffered from severe bone pain and developed paralysis of both lower limbs. Her laboratory tests were normal. A spine MRI revealed an abnormal signal in T8 and L5, and a hip MRI revealed an abnormal signal in the femoral head (Figure 1). We treated her with adjuvant radiotherapy, with a total dose of 30 Gy in 2.0 Gy daily. However, the bone pain lingered. Given that the involved sites in this case were mainly bone lesions and that multi-line chemotherapy including the R-CHOP regimen showed low efficiency, the patient was given repeated infusions of HD-MTX (MTX 3.2 g/m^2^, iv drip, day 1, administered every 21 days), followed by leucovorin (LV) rescue administration. The patient had an adverse reaction of neutropenic fever after receiving HD-MTX chemotherapy, which improved with symptomatic treatment. After 10 courses of HD-MTX treatment (six cycles of induced chemotherapy and four cycles of maintenance chemotherapy), the patient achieved complete metabolic remission, as confirmed by 18F-FDG PET/CT, and pain lessened. No therapy was applied to the patient after 2 August 2013. The efficacy evaluation showed complete remission during later follow-ups (Figure 2). During the last follow-up (February 2022), the patient showed a high quality of life, was free from pain, and walked smoothly. The treatment process of this case is shown in Figure 3.

## 3 Discussion

The differential diagnosis of bone lesions of the patient included chronic osteomyelitis, tuberculosis, primary bone sarcoma, leukemic infiltration, Ewing sarcoma, metastatic sarcomas, and carcinoma. Krishnan et al. (6) analyzed several cases and indicated that MRI was the most accurate method to detect lytic lesions, and lesions with a permeative pattern on radiographs were linked to soft tissue masses. Computed tomography (CT) scan was used to detect cortical erosion. The situation is highly suggestive of lymphomas such as solitary, permeative, or metadiaphyseal lesions with the periosteal reaction on plain radiographs and soft tissue involvement on MR images. In our case, the MRI scan and 18-F-FDG PET/CT demonstrated the tumor lesions were mainly concentrated in the vertebra.

Compared with patients with secondary bone lymphoma, those with PBL have a better prognosis, especially PB-DLBCL. Some studies revealed that the 5-year progress-free survival (PFS) and overall survival (OS) rates of patients with PB-DLBCL were 80 and 82–93%, respectively (4, 5), while the 5-year disease-free survival (DFS) rate for patients with systemic lymphoma with bone involvement was 44% (7).

Combined immunochemotherapy regimens consisting of R-CHOP are regarded as a standard-of-care curative treatment for DLBCL (8, 9). Previous therapy of PB-DLBCL is mostly based on the treatment of classical DLBCL. In the present case, a local hospital treated the patient with the R-CHOP regimen, which was unsuccessful. A retrospective report of 109 cases proved that chemotherapy alone or combination of chemotherapy and radiation was better than no treatment or radiation alone (10). Randa et al. (4) suggested that patients with PBL treated with R-CHOP-based chemotherapy, followed by consolidative radiation therapy (RT), showed excellent outcomes. The use of consolidative RT could improve 5-year PFS (88% RT vs. 63% no RT, *P* = 0.0069) and OS (91% vs. 68%, *P* = 0.0064) rates. The dose–response relation has not been determined yet. This study showed no difference between 30 and 35 Gy vs. >36 Gy; in our case, we used a dose of 30 Gy. The symptoms were partially relieved after radiotherapy, with no obvious adverse reactions.

High-dose methotrexate, as an induction strategy, is a conventional approach in patients with primary CNS lymphoma. The IELSG 14 study reported that multifocal bone diffuse large B-cell lymphoma (MB-DLBCL) patients with spine and skull involvement had a risk for central nervous system (CNS) relapse (11). The addition of HD-MTX to the CHOP/R-CHOP regimen significantly improved the prognosis of patients with high-risk DLBCL, irrespective of their risk for CNS relapse (12).

After the failure of multiple treatment lines, the prognosis of this patient with refractory PBL was very poor. MTX, a structural analog of folic acid, functions by inhibiting dihydrofolate reductase (DHFR), which converts dihydrofolate (DHF) to active tetrahydrofolate (THF) (13). THF is essential for DNA synthesis and cell growth, which is required for the biosynthesis of purines and thymidylate from serine. MTX is widely used in the treatment of many neoplasms, including lymphoma. HD-MTX with LV is used to treat non-Hodgkin lymphomas as a single agent in patients who have previously failed to respond to conventional chemotherapy (14). MTX has been examined in combination with chemotherapy in the treatment of bone tumor (15–17). Potent anti-metabolic and anti-proliferating actions are linked to the therapeutic application of MTX as an anticancer agent. Bologna et al. (18) showed that following MTX intramuscular administration in patients with rheumatoid arthritis (RA), MTX peripheral blood concentrations reached 0.0252 nmoles/ml. MTX concentrations in the trabecular and the cortical bone reached 0.292 and 0.286 nmoles/gm, respectively. A high MTX concentration can be found in the cortical and the trabecular bone (12), to some extent, which can explain the therapeutic effect of methotrexate on bone tumor. Based on these lines of evidence, we used HD-MTX as a trial and finally achieved surprising results. Leucovorin rescue is essential to minimize the risk of HD-MTX side effects (19).

In our patient, the compression of the spinal cord and nerve roots caused significant pain and progressive neurological dysfunction. Therefore, local radiotherapy is an effective approach to relieve spinal cord compression. The initial consideration of the HD-MTX regimen was due to the high concentration of MTX in the bone. Moreover, PBL involving the spine and skull involves the risk of central nervous system recurrence (11). MTX is the most widely used therapy for CNS prophylaxis in patients with DLBCL. Our patient was critically ill and urgently needed treatment, so we tried to the patient with a high-dose methotrexate treatment at last. However, the mechanism of radiotherapy combined with HD-MTX in the treatment of refractory PBL remains to be further confirmed.

## 4 Conclusion

To the best of our knowledge, this is the first time that HD-MTX has been used as a monotherapy for PBL. In our case, we used HD-MTX monotherapy after local radiotherapy, which was successful; this indicates that HD-MTX might be a salvage treatment for relapsed/refractory PB-DLBCL. The underlying mechanisms of HD-MTX monotherapy for PB-DLBCL remain unclear. This study included only one case; therefore, future studies including more cases are needed for further research and follow-up.

## Data availability statement

The raw data supporting the conclusions of this article will be made available by the authors, without undue reservation.

## Ethics statement

Ethical review and approval was not required for the study on human participants in accordance with the local legislation and institutional requirements. The patients/participants provided their written informed consent to participate in this study. Written informed consent was obtained from the individual(s) for the publication of any potentially identifiable images or data included in this article.

## Author contributions

ZW wrote first draft of the manuscript. WT had the idea for the manuscript. YT performed the literature search and data collection. JF approved the final version of the manuscript. All authors commented on previous versions of the manuscript and read and approved the final manuscript.
